# Evaluation of Apple Pomace Flour Obtained Industrially by Dehydration as a Source of Biomolecules with Antioxidant, Antidiabetic and Antiobesity Effects

**DOI:** 10.3390/antiox9050413

**Published:** 2020-05-12

**Authors:** Stanislava Gorjanović, Darko Micić, Ferenc Pastor, Tomislav Tosti, Ana Kalušević, Slavica Ristić, Snežana Zlatanović

**Affiliations:** 1 Institute of General and Physical Chemistry, P.O. Box 45, 11158 Belgrade 118, Serbia; dmicic@iofh.bg.ac.rs; 2 Faculty of Chemistry, University of Belgrade, Studentski trg 12-16, 11080 Belgrade, Serbia; fpastor@chem.bg.ac.rs (F.P.); tosti@chem.bg.ac.rs (T.T.); 3 Institute of Meat Hygiene and Technology, Kaćanskog 13, 11000 Belgrade, Serbia; anakalusevic@gmail.com; 4 Faculty of Medicine, University of Belgrade, Dr Subotića 8, 11000 Belgrade, Serbia; slavicaristic8@gmail.com

**Keywords:** apple pomace, antioxidant activity, dehydration, dietary fibres, diabetes, gluten-free flour, flavonoids, obesity, phlorizin, potassium

## Abstract

Apple pomace flour (APF) obtained at industrial scale level by the application of innovative technological process (dehydration (5 h, T ≤ 55 °C), grinding (300 µm)) was evaluated as a source of bioactive compounds with antioxidative, antiobesity and antidiabetic effects. Proximate composition, individual (HPLC–DAD–MS/MS) and total phenols (TPC) as well as flavonoids content (TFC), antioxidant (AO) activity (DPPH, ABTS, HPMC), water and oil holding capacity (WHC and OHC) of APFs obtained from apple pomace from mixed and individual apple cultivars grown conventionally and organically were compared. The effect of APF supplementation on the glycaemic status and glucose tolerance (oral glucose tolerance test (OGTT)) of C57BL/6J mice exposed to high-fat and sucrose diet was examined. High K content (4.2–6.4 g/kg), dietary fibres (35–45 g/100 g), TPC (4.6–8.1 mg GAE/g), TFC (18.6–34.6 mg QE/g), high water and oil holding capacity (4.7–6.4 and 1.3–1.6 g/g) were observed in the APFs. Content of major phenols (phlorizin, chlorogenic acid, quercetin), TPC and TFC correlated highly with prominent AO activity. APF supplementation lowered the increase of body weight gain and blood glucose, and improved glucose tolerance significantly. Health-promoting biomolecules, AO activity, functional properties and prevention of diet-driven glucose metabolism disorders pave the way to APF exploitation in human nutrition.

## 1. Introduction

Vast amounts of apple pomace (AP; peel, flesh, stem, core, seeds, juice residues) are generated annually, accounting for 25% of the original fruit mass in conventional juice processing. The world production in 2017/2018 was 77 million [1] and Serbian about 460,000 tonnes [2]. Phenols (catechins, procyanidins, phlorizin, phloretin glycosides, caffeic and chlorogenic acid, quercetin, cyanidin glycosides) and dietary fibres (DF; soluble pectins, β-glucans, galactomannan gums, non-digestible oligosaccharides, including inulin and insoluble lignin, cellulose and hemicelluloses) present in AP exhibit antioxidative, cardioprotective, antidiabetic, gastroprotective and antilipemic effects [3]. Despite numerous health benefits and a high potential for utilisation in nutraceuticals [4], this abundant and renewable natural resource is still underutilised. Processing without deterioration of biologically active compounds represents a major bottleneck for AP’s commercial utilisation in human nutrition and pharmaceuticals.

Drying is the most economically viable approach to stabilising perishable AP containing a high level of water (75–80 wt %), reducing its volume and lowering transportation costs. The method of moisture removal is an important factor affecting content, activity and retention of phenols, as well as functional properties of DF [5]. Common industrial practice is based on conventional methods such as drum drying and hot air as a drying medium. However, the high temperatures used cause degraded and compromised bioactivity of thermally sensitive compounds [6]. The drawbacks of conventional methods could be overcome by lowering drying temperatures. Freeze drying retains a higher level of bioactive compounds than conventional, vacuum oven and ambient air drying methods and maintains a whole set of functional properties (density, water and oil binding capacity, swelling capacity and glucose retardation index) [7]. However, it is time-consuming and too costly for industrial application [8]. Thus, there is still interest in the development of an economically and technically feasible technology for AP preservation at an industrial scale that will enable quick reduction of water content resulting in a stable product with low water content and activity and high content of biomolecules. Recently, a promising dehydration technology, adequate in terms of drying rate and utilised energy, was developed [9]. So far, there are no reports on the presence of biologically active compounds, including antidiabetic and antiobesity agents, in industrially dehydrated AP or information for its usage as a functional food for prevention of metabolic disorders, such as diabetes and obesity. Until now, most studies were focused on bioactive molecules presence in AP obtained or/and dried at laboratory scale as well as on its in vitro and in vivo effects and applicability. Despite its importance, investigations of industrially dried AP are scarce [10]. 

Thus, the aim of this study was to apply the newly developed dehydration technology to whole AP originating from mixed and individual varieties of both conventionally and organically grown apples at an industrial scale and to analyse the content and the activity of biomolecules present in apple pomace flour (APF) produced by grinding dehydrated AP. In addition, we examined its functional properties and its effect on metabolism, essential for further application in food and dietary supplement industries. Five samples of APF produced within the scope of the study were characterised in terms of mineral, dietary fibres (DF), phenols (TPC) and flavonoids (TFD) content as well as water and oil holding capacity (WHC and OHC), and subsequently compared to the most similar commercially available product. Individual phenolic compounds were identified and quantified by HPLC coupled to diode array and mass spectrometric detectors (HPLC–DAD–MS/MS). Antioxidant (AO) activity (radical scavenging capacity towards artificial radical species DPPH (2,2-diphenyl-1-picrylhydrazyl) and ABTS (2,2′-azino-bis(3-ethylbenzothiazoline-6-sulphonic acid) and reducing power (HPMC (HydroxoPerhydroxoMercury(II) Complex)) were determined as well. Regression analysis was performed to establish relationships between the analysed parameters and evaluate the contribution of phenol subclasses, as well as major individual phenols identified, to AO activity. An in vivo study was conducted to reveal the effect of APF supplementation on body weight management, glycaemic status and glucose tolerance of animals exposed to a high-fat and sucrose diet as well as standard diet. This was essential to confirm the efficiency of the technological process in terms of preservation of biomolecules in their physiologically active form, as well as the applicability of the produced APF in food fortification and dietary supplements.

## 2. Materials and Methods 

### 2.1. Materials

#### 2.1.1. Apple Pomace Flour Production

Five batch samples of APF were obtained by whole AP (pulp, peel, seeds and twigs) dehydration. No treatment other than squeezing of fresh fruit was used. AP was collected aseptically, immediately after squeezing and was dehydrated using a Solaris dehydrator (Solaris + Dehydrator, Serbia) [11] without any pre-treatment, for 4–6 h at 55 °C. The temperature and the humidity, being the key factors of the dehydrated products’ quality, were automatically fine-tuned and monitored by highly sophisticated software throughout the dehydrating process. With the dehydration process used, it was possible to quickly achieve low water activity required for high stability and long shelf life of the product, while allowing for premium quality in terms of colour and aroma. Dried AP was ground to a particle size below 300 microns and stored in multilayer paper sacks. APF obtained from AP provided by juice producer Fruvita, Smederevo, originated from mixed apple varieties (Idared, Jonagold and Golden Delicious in a random ratio; APF1), Idared (APF2) and Granny Smith (APF4) grown conventionally in the orchards of Smederevo district, while samples provided by organic juice producer Healthy Organic-Selenča originated from mixed apple varieties (Idared, Jonagold and Golden Delicious in a random ratio; APF3) and Red Delicious variety (APF5) grown organically in the South Bačka District, Vojvodina, Serbia. Apple egg (AE; Anti-Grain Foods LLC, Denver, CO, USA), declared as apple pomace powder with pectin added, was purchased. Water activity (aw) was determined by the aw meter Testo 650 (Testo AG, D-79853, Lenzkirch, Germany).

#### 2.1.2. Chemicals

All chemicals used were of analytical or HPLC grade. Standard solutions and blanks were prepared with ultrapure water (Thermo Fisher TKA MicroPure water purification system, 0.055 μS/cm). 

### 2.2. Elemental Analysis

APF samples (0.5 g) digested in concentrated nitric acid solution (5 mL) using a microwave oven (Milestone, Ethos 1, Terminal T640, Italy) were subjected to multi-element analysis by an ICP–OES spectrophotometer (Agilent, 5100). Prior to the analysis of macro- and microelements, sequential wavelength analysis was performed for every element, ensuring the selection of the most sensitive wavelength.

### 2.3. Determination of Dietary Fibre Content

Content of dietary fibres was determined according to AOAC Method 985.29 enzymatic–gravimetric method [12].

### 2.4. Determination of Total Phenol (TPC) and Flavonoid (TFC) Content

APF (100 mg) was extracted for 60 min with a 1:1 mixture of alcohol and water (1400 μL) at room temperature and centrifuged at 12,000 rpm for 10 min.

Total phenol content was determined according to the procedure reported by Singleton and Rossi [13]. An aliquot of APF extracts (0.25 mL) was mixed with Folin–Ciocalteu’s phenol reagent, diluted 10-fold (1.25 mL) and allowed to react for 6 min. Sodium carbonate solution (75 g L^−1^, 1 mL) was added and the mixture was shaken. After 2 h in the dark at room temperature, the absorbance was measured at 765 nm. The results were expressed as mg gallic acid equivalents per gram of sample (mg GAE g^−1^).

Total flavonoid content was determined according to the procedure reported by Zhishen and Mengcheng [14]. An aliquot of APF extracts (2.5 mL) was mixed with 150 μL of 5% NaNO_2_ solution; after 6 min, 10% AlCl_3_ (150 μL) was added, left to react for 5 min, after which 1 mL of 1 mol L^−1^ NaOH solution and 1.2 mL of distilled water were added. Absorbance was measured at 510 nm. The results were expressed as mg quercetin equivalents per gram (mg QE g^−1^).

### 2.5. Identification and Quantification of Phenolics by HPLC–DAD–MS/MS 

#### 2.5.1. Preparation of Samples

APF samples (1 g) were extracted with 20 mL of ethanol/water acidified (1 mL concentrated HCl/1000 mL water) mixture (70/30 *v*/*v*), for 24 h, with constant stirring. The obtained extracts were filtered, evaporated using a vacuum evaporator at 40 °C, dissolved in 5.0 mL of methanol/water (1/1 *v*/*v*) and stored at 4 °C until analysis. Prior to the analysis, the extracts were filtered through syringe filters with a pore size of 0.45 μm.

#### 2.5.2. Preparation of Standards

The 1000 mg/L stock solution mixture of all phenolic standards was prepared in methanol. Dilution of the stock solution with methanol yielded the working solution at concentrations of 0.025, 0.050, 0.100, 0.250, 0.500, 0.750 and 1.000 mg/L. Working and stock solutions were stable for 6 months stored in dark at 4 °C. Concentration ranges of all standards are given in Supplementary Table S1.

#### 2.5.3. Qualitative and Quantitative Analysis

Separation and quantification of polyphenols were performed using a Dionex Ultimate 3000 UHPLC system equipped with a diode array detector connected to a TSQ Quantum Access Max triple quadrupole mass spectrometer (Thermo Fisher Scientific, Basel, Switzerland) with the ion source in the form of electrospray ionisation (200 °C) in the negative mode (from 100 to 1000 m/z); triple quadrupole (UHPLC–DADMS/MS). The spray voltage was 5 kV and the temperature of the capillary was 300 °C.

Chromatography was performed at 40 °C on a Syncronis C18 column (100 × 2.1 mm, 1.7 μm particle size) as follows: 0.0–1.0 min 5% (B), 1.0−16.0 min from 5% to 95% (B), 16.0–16.1 min from 95% to 5% (B), then 5% (B) for 4 min, where 0.1% acetic acid in ultrapure water represents Eluent A and acetonitrile Eluent B. The flow rate was 0.300 mL min^−1^. For the purpose of quantification of polyphenols for each standard, the molecular ion and the two most intense fragments of the MS 2 spectra were recorded. Xcalibur software (version 2.2) was used to control the instrument. Phenol compounds were identified by direct comparison with commercial standards. The total amount of each compound was calculated from the corresponding calibration curve and expressed as mg kg^−1^. 

### 2.6. Determination of AO Activity and Relative Antioxidant Capacity Index (RACI)

#### 2.6.1. Radicals Scavenging

DPPH scavenging ability of extracts was determined according to the slightly modified procedure reported by Kaneda et al. [15]. An aliquot of the sample (0.2 mL) was mixed with 2.8 mL of DPPH solution (mixture of 1.86 × 10^−4^ mol L^−1^ DPPH in ethanol and 0.1 mol L^−1^ acetate buffer (pH = 4.3) in 2:1 volume ratio). After 40 min at room temperature in dark, absorbance was measured at 525 nm. 

ABTS scavenging ability was determined by the procedure reported by Re et al. [16]. A 30 μL volume of each diluted sample was mixed with 3 mL of the ABTS solution. Absorbance was measured at 734 nm after 6 min. 

The results of DPPH and ABST were expressed as mmol Trolox equivalents per gram of sample (mmol TE g^−1^).

#### 2.6.2. Reducing Activity

HPMC (HydroxoPerhydroxoMercury(II) Complex) assay was used as described [17]. A dropping mercury electrode (DME) with a programmed dropping time of 1 s as a working electrode, a saturated calomel electrode (SCE) as a reference and a Pt-foil as an auxiliary electrode were used. A 100 µL volume of 1.00 M H_2_O_2_ was added into an electrolytic cell with 19.9 mL of Clark and Lub’s buffer (pH 9.8). Before the first and after each addition of five aliquots of 100 µL of APF extracts, nitrogen was purged and *i-E* curves recorded. The decrease of the initial anodic current of HPMC, observed upon each addition, was calculated and plotted versus the amount of extract added. Results were expressed as anodic limiting HPMC current decrease per mg of APF extracted (% mg^−1^). 

#### 2.6.3. Relative Antioxidant Capacity Index (RACI)

RACI was calculated based on the results obtained employing all AO assays mentioned, by assigning equal weight to each of them as described [17]. A standard score was calculated according to the following equation:

Standard score = *(x − μ)/σ* (1)

where *x* is the raw data, *μ* is the mean and *σ* is the standard deviation. The standard scores of a sample for different assays, when averaged, gave a single unitless value termed RACI.

### 2.7. Determination of Water and Oil Holding Capacity and Solubility of APF

For water holding capacity (WHC) and oil holding capacity (OHC) determination, 1 g of APFs was mixed with 30 mL of distilled water and 10.0 mL of sunflower oil, respectively, and left for 24 h at room temperature. After centrifugation at 3600 rpm for 20 min, the supernatant was removed and the weight of the residue measured. Results were expressed as the weight of water bound or oil retained per gram of sample (g/g). The water holding capacity was corrected by the addition of a mass of the presumably soluble constituents removed to the measured weight increase after the soaking and centrifugation of the powder (WHC-c). The solubility of APF samples and AE was determined as described previously [18].

#### 2.7.1. Animals

Eight-week-old male C57BL/6J mice, with initial body weights of 18–22 g, were randomly distributed among four groups (*n* = 8 mice/group) and housed in standard cages in a room with a 12 h light–dark cycle at a temperature of 22 ± 3 °C. The study was performed according to the regulations and standards of the national (Serbian) Law on the Experimental Animal Treatment and European Directive 2010/63/EU (European Convention for the Protection of Vertebrate Animals used for Experimental and other Scientific Purposes) and approved by the Ethics Committee for experimental animals welfare of the Faculty of Agriculture, University of Belgrade and Ministry of Agriculture, Forestry and Water Management (Process 323-07-00617/2017-05). 

#### 2.7.2. Diets 

Two groups were exposed to a high-fat (20% lard) and sucrose (20% sucrose as drinking solution) diet without (high-fat and sucrose diet (HFSD)) and with the addition of 10 mg APF per day (HFSD + APF10) while the control groups were fed with standard pellet rodent diet without (SPRD) and with the addition of 10 mg APF per day (SPRD + APF10) and water for 150 days. In standard rodent food (Veterinary Institute Subotica, Serbia) based on corn, wheat, barley and soybean protein ((20%), fat (5%), carbohydrates (40%), cellulose (8%), Ca (1%), K (1%), P (0,5%), lysine (0.9%), methionine + cystine (0.9%), Na (0.25%), ash (10%) and water (13%), as well as A, D3, B12, E, K3, B1, B2, B6, C, Ca pantothenate, niacin, folic acid, choline, Fe, Zn, Mn, J, Se and artificial antioxidant (BHT)) 5 g APF per kg was incorporated before pelleting. Pellet size was about 2 cm length and 2 g weight. Standard rodent food with and without 6.25 g APF per kg was mixed well with pork lard (Trlić Company, Serbia) in an 80:20 ratio to be used in HFSD diet without and with 5 g APF/kg. Average daily intake (2 g per mouse) contained approximately 10 mg of APF per mouse. The calorie content of each diet was calculated using the following specific energy factors: 9 for fat, 4 for carbohydrates and protein and 2 for dietary fibre.

All groups had access to feed and water ad libitum. The activity, behaviour and general health of the mice were monitored daily while feed and water (SPRD) or sucrose solution (HFSD) intakes and body weight were recorded weekly. All animals looked perfectly healthy and their behaviour was normal on intentional standard provocation tests. No neurological misbehaviour or abnormal reactions with respect to food or water were observed. At the end of the experiment, all animals were anaesthetised with intravenous thiopental, sacrificed and pathoanatomical examination was performed. Macroscopic examination of organs and tissues (liver, spleen, kidney, stomach, small intestine, lungs and heart) did not show any pathological changes.

#### 2.7.3. The Glycaemic Status 

The glycaemic status was monitored throughout the experiment period, on a monthly basis. Glucose was measured in fresh blood collected from the tail vein (one drop of full blood ~20 μL) on a biochemical analyser, the A15 automatic analyser from BioSystems (BioSystems S.A., Barcelona, Spain) with standard glucose determination reagents (phosphate 100 mmol/L, phenol 5 mmol/L, glucose oxidase >10 U/mL, peroxidase >1 U/mL, 4-aminoantipyrine 0.4 mmol/L, pH 7.5) using glucose oxidase/peroxidase method. Results were expressed in mmol/L.

#### 2.7.4. Oral Glucose Tolerance Test (OGTT)

Oral glucose tolerance test (OGTT) was performed within the last week of the diet. Overnight-fasted mice received a glucose solution (2 g/kg) by gavage. Blood was collected from the tail vein (one drop of full blood ~20 μL). Glucose concentration was determined prior and 30, 60, 90 and 120 min after glucose administration. Results were expressed in mmol/L. The area under the curve (AUC) was calculated using the trapezoidal rule. 

### 2.8. Statistical Analysis

XLSTAT (version 2014.5.03, Addinsoft, New York, NY, USA), analysis and statistics add-in for MS Excel, was used for statistical analysis of data. The results were expressed as means ± standard deviation (SD). All data were analysed by one-way ANOVA, except OGTT and relative body weight gain data, where a two-way repeated-measures ANOVA was used. In all cases where *p*-values of the ANOVA showed that there was a significant effect of any factor (*p* < 0.05), post-hoc Tukey’s honest significant difference (HSD) test was used to examine significant differences between means. The relationship between the parameters was determined by Pearson’s correlation analysis.

## 3. Results and Discussion

### 3.1. Production of APF 

Five samples of apple pomace flour (APF) were obtained from whole apple pomace (AP; peel, pulp, stems and seeds) applying a recently disclosed technological process [9] at the industrial scale. The dehydration, conducted in a closed system, had three distinct phases: (1) heating of wet AP, until constant temperature of up to 55 °C was reached, (2) drying at constant temperature while humidity decreased, (3) removal of residual moisture and cooling of dehydrated AP. Moisture of 4–6 wt % and water activity (aw) of 0.2–0.3 were achieved after 4–6 h of dehydration. Over 24 h was required to achieve the same effect by forced air and freeze drying [7,19,20]. The low aw achieved enabled grinding of dehydrated AP to a particle size of less than 300 µm and production of APF. Stability of APF at storage temperatures was studied by thermal analysis. The temperature of glass transition (Tg) of APF was well above storage temperatures [21]. High Tg was related to phytochemicals stability in dried apple products [22]. Both low aw and high Tg indicated a long shelf life of the produced APF. The produced flour with intense apple flavour was easy to preserve, transport and finally use as a food ingredient [18]. The obtained APF samples and the most similar commercially available product apple egg (AE), based on AP powder mixed with pectin, were characterised in parallel in terms of mineral and DF content, TPC and TFC.

### 3.2. Composition of APF 

#### 3.2.1. Mineral Content 

The concentrations of potassium, calcium, sodium, magnesium, copper and zinc in APF (K 4228 ± 76 to 6398 ± 115, Ca from 455 ± 8 to 724 ± 12, Na from 394.0 ± 6.7 to 715.0 ± 12.0, Mg from 239 ± 5 to 510 ± 10, Cu from 1.80 ± 0.37 to 2.30 ± 0.35 and Zn from 0.59 ± 0.09 to 8.91 ± 1.34 mg/kg) were found to be higher than in whole apple. Unlike flours from cereals and pseudo-cereals, APF does not contain phytic acid that decreases the bioavailability of Ca, Mg, Zn and Cu. Adequate potassium intake of 3.5 g/day for adult men and women is rarely met in the typical Western diet. APF’s potential as a potassium intake increasing additive was indicated by four- to seven times higher potassium content than in rice flour (974 mg/kg) and three to four times higher than in wheat (1500 mg/kg) and corn flour (1487 mg/kg) [23]. Potassium acts as the main enzymatic cofactor in acid-base balance maintenance, reduces the risk of stroke and coronary heart disease but also plays an essential role in the functioning of nerves and muscles and in diabetes prevention [24]. Metadata from several relevant studies were subjected to nonlinear dose–response analysis and protective effect of adequate potassium intake on obesity and metabolic syndrome was demonstrated [25].

#### 3.2.2. Content of Dietary Fibres

Proximate composition of APF determined by Zlatanovic et al. [21] showed that the content of proteins (3.2 ± 0.3 to 5.8 ± 0.3 g/100 g), fats (1.0 ± 0.1 to 2.7 ± 0.2 g/100 g) and total carbohydrates (44.2 ± 4.2–49.5 ± 6.1 g/100 g) was in accordance with the previously reported data [3]. Components that are indigestible for the human enzyme system represent a major part of AP. Their content is variable, depending on apple cultivar and technological process of juice extraction. The range of total dietary fibre (DF) content in APF (35–45%) was found to be in accordance with previously reported data for AP [4] and with the value determined for AE (44 ± 4 g/100 g). Both wheat (3.45 ± 0.01 g/100 g) and gluten-free flours from oats (0.43 ± 0.15 g/100 g), buckwheat (4.05 ± 0.40 g/100 g), maize (2.18 ± 0.11 g/100 g) and rice (2.62 ± 0.45 g/100 g) are very low in DF [23]. APF from mixed varieties of apples (APF1), produced in the highest quantity in Serbia (APF1), possessed 100, 20, 17 and 12.5 times higher content of DF (45.0 ± 0.7 g/100 g) than oat, maize, rice and wheat flour, respectively. Therefore, APF has huge potential to compensate for the lack of DF in confectionery and bakery products, particularly those that are gluten-free. DF content in APF is high even in comparison to berries and whole-grain cereals. In comparison to DF from cereals, AP fibres are characterised by a better ratio between soluble and insoluble portion, higher presence of associated bioactive compounds such as antioxidants, and absence of anti-nutritional factors such as phytic acid [26]. Improvement of the gastrointestinal tract (GIT) function, as well as antioxidative, cardioprotective, antidiabetic and antilipemic effects, were related to a DF-rich diet. The hypoglycaemic potential of apple DF and a beneficial effect of high intake in patients with type 2 diabetes were documented [3].

#### 3.2.3. Total Phenol and Flavonoid Content

Phenol compounds present in the peel are major apple AOs. Phenols are the predominant ingredients in apple seeds as well [27]. Phenol content in whole apple, seeds and peel mostly varied depending on the cultivar, whereas their content in AP is also influenced by juice production technology, further processing and storage conditions [6,28,29]. Content and preservation of phenols in dried AP are also associated with drying conditions [4,7,20]. Since the same process of juice extraction and flour production was employed, differences in TPC and TFC of different APFs (Table 1) can be related only to the differences in apple cultivars. Significantly higher TPC was found in APF from mixed cultivars with Idared predominant (APF 1 and 3) or single (APF2) than in APF 4 and 5 from Granny Smith and Red Delicious or AE. The TPC (7.7 ± 0.3 mg GAE/g) in APF1 was found to be 60, 5, 8 and 1.5 times higher than in wheat (0.13 ± 0.02 mg/g ), rice (1.42 ± 0.24 mg/g), maize (0.98 ± 0.10 mg/g ) and buckwheat (4.66 ± 0.22 mg/g) flour determined by Hager et al. [23], respectively. 

Upon 12 months of storage in adequate conditions, no significant decrease of TPC and TFC was observed (*p* < 0.05). Good retention of phenols during APF storage in common conditions, enabled by low aw values achieved by dehydration at an industrial level, is in good agreement with the already reported thermal stability, i.e., high temperature of glass transition [21]. This result supported the suggestion given by Laveli [30] that appropriately dried AP can be used in the development of new food matrices with maximum retention of biomolecules. Good retention of phenols present in AP dehydrated at laboratory scale (air drying at 60 °C) [5], as well as in apple products with low aw [31], was shown.

Phenol-rich foods represent a promising approach to type 2 diabetes management. The role of apple polyphenols on the management of diabetes has been reviewed by Rana and Bhushan [32]. Modulation of activity of digestive enzymes, α-amylase and α-glucosidase, resulting in a lower glycaemic index of food, i.e. a lower rate of carbohydrate digestion and glucose adsorption, is one of several underlying mechanisms. Anti-diabetic effects and mechanisms of action of flavonoids [33] and phenolic acids, affecting also most of the leading aspects of obesity including diabetes, insulin resistance, hyperglycaemia, hyperlipidaemia and adipocyte dysfunction and inflammation [34] were investigated.

### 3.3. Identification and Quantification of Individual Phenolic Compounds Present in APF

Composition and concentrations of the major phenols identified in APF 1–5 and AE are shown in Table 2. Individual phenolic compounds were grouped into corresponding phenolic classes (dihydrochalcones, flavanols, flavanones, flavonols and flavonol glycosides, flavanonols, coumarins) and their levels were calculated from the sum of all identified compounds.

Total phenol content quantified by HPLC varied from 948.6 (APF3) to 560.5 mg kg^−1^ (APF5) with variation coefficient 22.5%. APF1 and APF3 showed the highest content in all phenols analysed, followed by APF2. APF2 originated from a single cultivar, Idared, while APF1 and APF3 were obtained from mixed cultivars with Idared predominant. The lowest content was in APF4 and APF5, originating from single cultivars Granny Smith and Red Delicious, respectively. The finding that TPC determined by FC was higher than the sum of individual phenols quantified by HPLC can be explained by the interference of various substances other than phenols (organic acids, residual sugars, amino acids, proteins and other hydrophilic compounds) in the FC assay, various responses of individual phenols, presence of only low molecular weight phenols in analysed extracts [27,35], as well as missing values of unidentified polyphenols by HPLC/MS. However, the relationship between TPC determined by FC and calculated based on HPLC was found higher in this study (r 0.89) than in the study focused on AP from various apple cultivars commonly grown in Serbia (*r* = 0.72) [35].

Among individual polyphenolics identified, phlorizin and chlorogenic acid (CGA) were predominant. Phlorizin content varied from 111.2 (APF 4) to 227.3 mg kg^−1^ (APF3) with variation coefficient 25.1%, accounting for 18–29% of the sum of all identified phenols. Variations in phlorizin content in AP as well as in apple peel and seed were shown to be associated mostly with genetic variety [27,35]. Variations in CGA content in APF samples were higher (variation coefficient 38.7%). Its content ranged from 89 mg kg^−1^ (APF4) to 308.3 mg kg^−1^ (APF 3), accounting for 14.4% to 32.5% of the total sum of identified phenols. Both phlorizin and CGA were shown previously to be the most stable among apple phenols. The minimal rate of apple phenol degradation was observed at the lowest aw values (0.30) [31]. At higher aw (0.75), the degradation occurred in the following order: procyanidin B2, cyanidin 3-*O*-galactoside, epicatechin, quercetin 3-*O*-galactoside, CGA and phlorizin [31]. 

Quercetin is present as aglycone and glycoside, galactoside (hyperin) and rhamnoside. The content of glycosides accounted from 18.3% to 21.7% for hyperin and 6.1% to 13.5% for quercetin-3-*O*-rhamnozide, while quercetin aglycone accounted for less than 2% of total phenols. Presence of a wide variety of quercetin glycosides was observed in the apple cultivars previously, as well as the prevalence of galactoside over rhamnoside [36].

### 3.4. Antioxidant Activity 

Apple peel phenols contributed more to the total AO activities of whole apple than apple flesh phenols. Only 3−10% of the overall AO activity of an apple remains in the apple juice [7,35]. For a more comprehensive and reliable comparison between all analysed samples, three in vitro assays were carried out to evaluate AO activity of APF samples both after their production and after a year of storage. Radical scavenging capacity towards two artificial radical species and reducing power measured based on the decrease of anodic limiting current of HPMC of APF1–5 and AE are shown in Table 3, along with Relative AO Capacity Index (RACI) calculated by assigning equal weight to each AO assay result. 

Scavenging effect towards the ABTS revealed prominent AO activity of APF1–3, approximately three times higher than AE. According to the DPPH assay, there was a significant difference between APF1–3 and AE as well. Electrochemical HPMC assay showed 1.5 to 2 times higher AO activity of APF1–3, and significantly higher activity of APF4 than AE. RACI confirmed APFs superiority over AE. As seen in Table 3, the highest RACI value has been found in APF3. No significant change of AO activity was observed after storage. Both the AO properties related to phenol content and good retention of AO activity during storage are important features of APF. 

Numerous reports reveal that incorporation of AP in food formulations upgraded AO activity preventing or delaying oxidative change, i.e., lipid oxidation in the food matrix. Apple phenols, the major carriers of AO activity, exhibited stronger phospholipid protective capacity than BHT and their effect in enhancing the oxidation stability of meat products was shown [37]. As a natural alternative to artificial preservatives, APF could be used in various food formulations as well. Recently, it was incorporated in confectionery and dairy products. Improved storability of cookies with 25%, 50% and 75% of wheat flour replaced by APF was reported, indicating the contribution of APF to shelf life extension [18].

### 3.5. The Correlation Analysis: Individual Phenol Contribution to Total AO Activity

TPC and TFC content correlated well with the results of all three AO assays (Table 4). The distinct correlation obtained for APF, as well as previously reported for AP [35], proves that phenolic compounds contribute directly to AO activity. A stricter correlation was observed between TPC and AO determined by radical scavenging than by HPMC. Higher correlation of HPMC with TFC than with TPC could be explained by a better response of flavonoid than phenolic acids, as noticed previously for plant extracts [17]. 

Among phenol subclasses, the correlation between DPPH, ABTS and HPMC and total content of cinnamic acids (0.95, 0.88 and 0.81) and dihydrochalcones (0.87, 0.86 and 0.74; *p* < 0.05) was the highest, indicating their contribution to total AO activity. In the mixture of phenols present in apple, apple peel or AP, the relative contribution of each antioxidant to the total AO capacity was investigated previously [35,36,38,39,40,41]. Either CGA, or flavonoids, such as quercetin and phlorizin, were most consistently reported as the main contributors to total AO activity. 

CGA content quantified in APF correlated with correlation indices of 0.94, 0.86 and 0.80 with DPPH, ABTS and HPMC, respectively. CGA was reported to be the most significant contributor to ABTS scavenging activity and total reducing power (FRAP) of powders of five varieties of apple cultivars obtained by freeze drying [38]. Besides CGA and quercetin, high AO activity of apples was also related to protocatechuic acid [39]. 

Phlorizin content in APF correlated 0.85, 0.85 and 0.74 with DPPH, ABTS and HPMC, respectively. Phlorizin present in the Golden Delicious variety displayed the highest contribution to AO activity determined by four different AO assays [41]. According to Diñeiro García et al. [40], AO activity of AP can be predicted by phlorizin, procyanidin B2, rutin + isoquercetin, protocatechuic acid and hyperin content. 

### 3.6. Functional Properties of APF 

**Figure 1:**
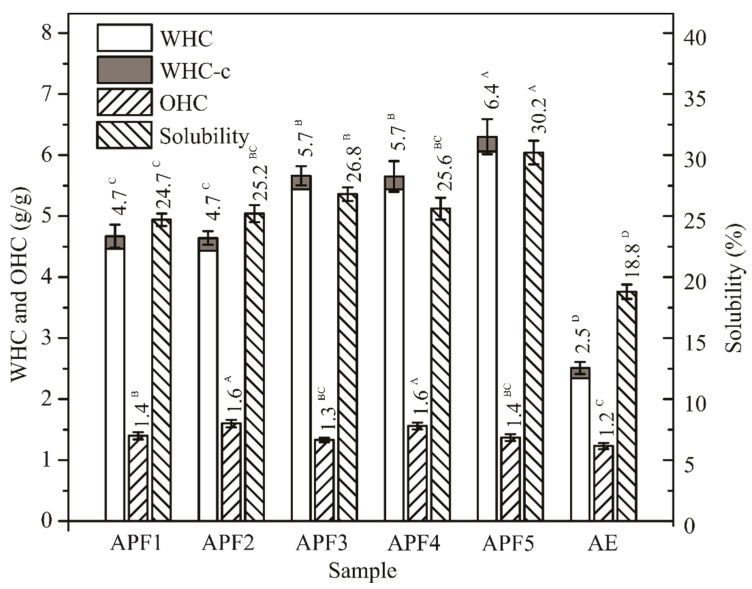
Water holding capacity (WHC) corrected for contribution of the soluble part of the APF (WHC-c), solubility and oil holding capacity (OHC) of APF samples in comparison to most similar commercially available products (AE). Data were subjected to one-way ANOVA (between-subjects factor: type of APF (*n* = 3); six levels: APF1, APF2, APF3, APF4, APF5 and AE; degree of freedom for both parameters was 5; for WHC *F* = 135.4 and *p* < 0.001, for OHC *F* = 20.9 and *p* < 0.001), different superscripts within the same parameter indicate a significant difference of means, according to Tukey’s HSD test (*p* < 0.05).

High WHC-c (4.7–6.4 g/g) and OHC (1.3–1.6 g/g; Figure 1) was expected, due to high content of DF present in APF. Since it was produced from minimally processed AP, dehydrated under very gentle conditions, structural changes of the cell wall polysaccharides did not occur. 

Determined values were 1.9–2.6- and 1.1–1.3-folds higher for APF samples than for AE, respectively, and much higher in comparison to cereal and pseudo-cereal flour. Functional properties of APF indicated its potential in fortifying and development of DF-rich and low-calorie food. High water holding capacity related to the presence of soluble dietary fibres can help increase matrix viscosity at GIT level. Gel formed might protect starch from the amylolytic activity of digestive enzymes and release of the free glucose resulting in a reduced glycaemic response. The decrease of the glycaemic index of functional food enriched with AP was reported recently [42]. The direct effect of insoluble DF related to OHC on postprandial glucose was not observed. However, insoluble DF affect gut transition time and were also associated with reduced diabetes risk [43]. As a promising food additive, APF could be incorporated in various food products, such as meat products, custards and soups to enhance thickening and viscosity and in baked products to improve freshness and handling, while at the same time reducing glycaemic index [42]. The impact of APF addition on functional properties of cookies enriched with 25%, 50% and 75% of APF was reported [18]. 

### 3.7. Effect of APF Presence in High-Fat and Sucrose Diet on Glucose Metabolism and Body Weight Management 

Evidence of AP influence on digestion and metabolism, lipids homeostasis and diabetes type II was summarised recently [4,44]. The content of various bioactive ingredients, abundant also in APF, can be linked to lower diabetes type II and cardiovascular diseases risks. In addition to DF, higher intake of major APF phenolics was associated with a decreased risk of some chronic diseases, including diabetes type II [32,45]. Previous studies confirmed the antidiabetic and antiobesity effects of major phenols identified in APF as well. Phlorizin improves hyperglycaemia by blocking renal glucose resorption and intestinal glucose absorption through inhibition of the sodium–glucose symporters. Its effect in preventing diet-induced obesity, hepatic steatosis, inflammation and fibrosis, as well as insulin resistance, was confirmed recently [46]. The effects of CGA on glucose tolerance, insulin sensitivity, hepatic gluconeogenesis, lipid metabolism and skeletal muscle glucose uptake were also shown. Anti-diabetic and anti-lipidemic effects of GCA are mediated by AMPK activation [46]. CGA promoted body weight loss, reduced plasma lipid levels and altered mRNA expression of lipogenesis and lipolysis-related genes in adipose tissue. Amelioration of diet-induced gut microbiota dysbiosis by CGA is proposed to contribute to its beneficial effects [46]. Mechanisms of quercetin antidiabetic activity involve the inhibition of intestinal glucose absorption, insulin-secretory and insulin-sensitising activities as well as improved glucose utilisation in peripheral tissues [46]. The inhibitory effects of quercetin in adipogenesis and inflammation were investigated using a mouse model and its effect in the management of metabolic disorders by regulating obesity and obesity-induced inflammation was demonstrated [47]. Quercetin reduced body weight, and suppressed expression of adipogenic, lipogenic and inflammation-related cytokines [47].

APF1 was chosen to be used in an in vivo study examining potential benefits of APF on glycaemic status, since it was the most abundant sample, rich in antidiabetic agents. The effect of adding of 10 mg of APF per day (0.5% w/w) to a high-fat and sucrose diet (HFSD) on glycaemic status, body weight gain and glucose metabolism in oral glucose tolerance test (OGTT) upon 150 days of diet was followed and compared with the effect of HFSD and standard pellet rodents diet (SPRD) with and without the addition of APF. Glycaemia, body weight gain, daily food, water and energy intake, as well as food efficiency ratio calculated as body weight gain (BWG) per food intake (FER1) and per energy unit (FER2), and area under OGTT curve, are shown in Table 5. The group fed a high-fat and sucrose diet (HFSD) showed a statistically significant (*p* < 0.05) increase in glycaemia (39.3%) in comparison to the control group (SPRD), while BWG was not statistically different. In the group fed HFSD supplemented with APF, no increase in glycaemia in comparison to SPRD occurred. In comparison to the HFSD group, glycaemia in HFSD-APF10 decreased by 38.7%. In groups fed both HFSD and SPRD with APF, BWG was significantly suppressed in comparison to diets without APF (60.7% and 39.2%, respectively). Both BWG and FER1 significantly differ between diets with and without APF. Daily food intake in the HFSD groups was very close but lower with a statistically significant difference at *p* < 0.05 than in SPRD groups while energy intake was more than 2.5 times higher. FER1 and FER 2 were higher in HFSD and SPRD groups than in their counterparts’ supplemented with APF. The difference in FER1 between HFSD and SPRD was not found statistically significant, as well as the difference between their counterparts supplemented with APF but FER1 of HFSD-APF and SRPD-APF were reduced 2.6 and 1.7 times in comparison to HFSD and SPRD, respectively. A similar reduction of FER 2 was noticed. 

Most animal studies conducted so far were short term (4–10 weeks) and doses of AP were much higher, usually from 5% to 20% *w*/*w*, than the dose applied here (0.5% *w*/*w*) or achievable by humans. Five-week-long supplementation with 10% (*w*/*w*) hot-dried AP (laboratory scale) suppressed BWG (34%) and improved lipid profiles in rats with high-fat, diet-induced obesity [48]. Administration of apple polyphenols showed a protective effect against BWG and fat deposition, and improved glucose tolerance in Wistar rats as well. AP was proposed to exert its antiobesity effects through the regulation of genes involved in adipogenesis, lipolysis and fatty acid oxidation [49]. The higher faecal lipid percentage was observed in Wistar rats supplemented with AP (68.8 g/kg) than orange bagasse (99.6 g/kg) or passion fruit peels (86.2 g/kg) [50]. Parameters of metabolic syndrome and atherogenic progression were monitored in mice exposed to a fat diet with fresh, sun-dried and dehydrated apple peel (20% *w*/*w*) for 43 days. The addition of fresh peels led to a significantly more intense lowering effect on glycemia and BWG (134 ± 30 mg/dL, 6.2 ± 0.5 g) than addition of dehydrated (294 ± 28 mg/dL, 6.7 ± 0.6 g) and sun-dried apple peel (278 ± 28 mg/dL, 6.0 ± 0.6 g), in comparison to HFD (343 ± 4 mg/dL, 7.5 ± 0.4 g) and standard diet (311 ± 10 mg/dL, 6.9 ± 0.1 g) [51]. A much more pronounced effect of APF added in significantly lower percentage (0.5% *w*/*w*) on glucose level and BWG was observed.

**Figure 2:**
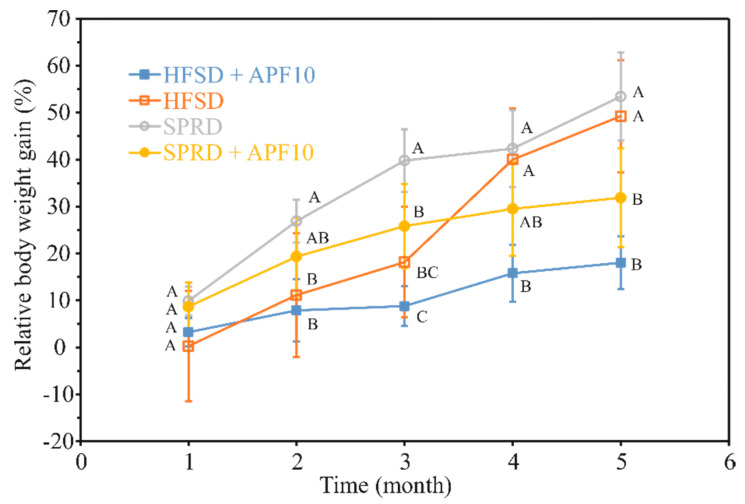
Relative body weight gain during 5 months exposure of mice to HFSD and SPRD with and without APF addition. Data were subjected to two-way repeated-measures ANOVA (between-subjects factor: feed treatment (*n* = 8); four levels: HFSD, HFSD + APF, SPRD and SPRD + APF, degree of freedom was 3, *F* = 9.8 and *p* < 0.001; within-subject factor: time; five levels: 1, 2, 3, 4 and 5 months; degree of freedom was 4, *F* = 202.3 and *p* < 0.001; interaction “feed treatment × time”; degree of freedom was 12, *F* = 16.0 and *p* < 0.001); different uppercase letters within the same time indicate a significant difference of means, according to Tukey’s HSD test (*p* < 0.05).

In order to get a better insight into the impact of long-term APF supplementation on weight management, BWG was followed monthly during the entire period that the animals were exposed to HFSD and SPRD diet supplemented with 10 mg of APF (Figure 2). Differences in BWG in the HFSD + APF10 and SPRD + APF10 groups in comparison to the HFSD and SPRD groups became more prominent. Due to the lowest food intake at the beginning of exposure, BWG increase in the HFSD group was lower but eventually caught up with the SPRD group. At the end of the period considered, no significant difference in BWG between the SPRD and HFSD groups was noticeable. By far the lowest BWG was noticed in the HFSD-APF group during the entire period considered. Prominent effects of APF presence on BWG reduction were observed in the SPRD-APF group, in comparison to the SPRD group, as well. 

Recent findings lead to a hypothesis that gradual accumulation of fat in the liver and pancreas caused beta-cell differentiation and loss of specialised function. Regulation of consequent hyperglycaemia was achieved by substantial weight loss that led to removing the excess fat from liver and pancreas [52]. Having in mind considerable implications of obesity prevention for personal and national health APF can be considered of particular importance as a natural antiobesity agent that can be produced at industrial scale level by the application of already developed technology.

**Figure 3:**
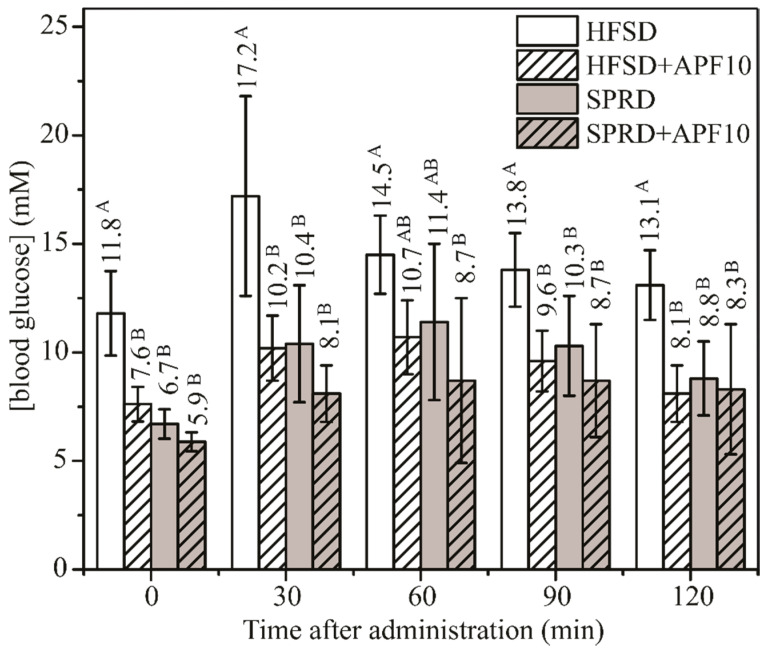
Plasma glucose concentrations in oral glucose tolerance test in mice after 150 days exposure to HFSD and SPRD with and without APF addition. Data were subjected to two-way repeated-measures ANOVA (between-subjects factor: feed treatment (*n* = 8); four levels: HFSD, HFSD + APF10, SPRD and SPRD + APF10, degree of freedom was 3, *F* = 16.6 and *p* < 0.001; within-subject factor: time; five levels: 0, 30, 60, 90 and 120 min; degree of freedom was 4, *F* = 14.9 and *p* < 0.001; interaction “feed treatment × time”; degree of freedom was 12, *F* = 1.4 and *p* = 0.182); different uppercase letters within the same time indicate a significant difference of means, according to Tukey’s HSD test (*p* < 0.05).

The influence of the chosen dose of APF on glucose metabolism in OGTT was investigated as well. As highly sensitive and specific in detecting glucose intolerance OGTT was performed within the last week of diet. Improved glucose metabolism in the OGTT (Figure 3) confirmed that APF supplementation in the amount of 0.5% *w*/*w* led to better glucose tolerance in animals exposed to HFSD. The values of the area under the curve (AUC) for HFSD (29.0 ± 3.6 mMh) and HFSD + APF10 (19.2 ± 2.0 mMh) were significantly different. Their ratio of 1.5 indicated significant improvement of glucose tolerance in the group exposed to HFSD supplemented with APF. During the entire glucose challenge, the difference between SPRD and SPRD+APF10 was observable but not statistically significant, as well as the difference between AUC values (19.9 ± 4.5 and 16.3 ± 3.7). 

A daily dose of 0.5 g of APF per kg of body weight or about 35 g per person of average body weight (70 kg) contains approximately a half of recommended daily intake of DF whose intakes higher than 25 g per day (adequate for normal laxation) are associated with improved BW maintenance and a reduced risk of diabetes type II [53], and significant amount of total phenols (270 mg GAE) and flavonoids (870 mg QE), including 7.5 mg of phlorizin, known as an antihyperglycemic and antihyperlipidemic agent in diabetes and obesity [54], as well as 7.8. mg of CGA and 10 mg of quercetin. Having in mind that Europeans consume 0.7–7.5 mg/d phlorizin from natural sources [55], it could contribute significantly. Both quercetin and its derivatives, and CGA present in 35 g of APF represent about half part of average daily intakes of Chinese and Japanese (20 and 15 mg per day, respectively) [56,57] while 150 mg potassium represents 1/20 of recommended daily intake for adults. Although dose estimated based on body weight, has been found close to doses applied in most human trials conducted until know (around 25 g of AP per day), more than 10 times lower translated dose was obtained using allometric scaling for dose conversion from mice to human proposed for drugs [58] (approx. 3 g/d). Throughout, the contribution of a translated dose of approx. 3 g of APF to a daily intake of DF is not a remarkable, presence of DF might be of importance, especially having in mind physiological function (the transportation of antioxidants through GIT) [59]. About 50% of AOs, mainly phenols, traverse the small intestine linked to DF. AP was already suggested to be used in the development of novel matrices with maximal retention of phytochemicals, particularly phlorizin, CGA and quercetin [30], as well as a carrier able to increase remarkably bioavailability of phytochemicals providing protection against degradation during in vitro digestion [60]. Thus, DF might increase the bioavailability of antidiabetic and antiobesity agents of phenolic origin. DF and phenolics from apple concentrate were already shown to be more effective together than separately on metabolic parameters related to the metabolic syndrome [61]. Positive effects on body weight management, glucose level and glucose tolerance indicated APF’s potential as a dietary supplement and confirmed, also, that the applied technological process provided preservation of biomolecules in their physiologically active form.

## 4. Conclusions

The gentle and fast technological process, applied to minimally processed apple pomace—a by-product from the food industry—allowed for the production of a nutrient-dense apple pomace flour with a high content of bioactive phytochemicals with good retention, high AO activity as well as good functional properties such as WHC and OHC related to the presence of soluble and insoluble DF. Thus, APF produced by dehydration at an industrial scale could be used as an efficient food fortifier that can bridge the DF and phytochemicals deficit in the modern diet, particularly prominent in the gluten-free diet. Antioxidant, antidiabetic and antiobesity effects of APF allowed use as a dietary supplement beneficial in preventing radical-induced oxidative stress and diet-driven glucose metabolic disorders. Long-term (five months) supplementation with APF (0.5% *w*/*w*) was shown to decrease glycemia (approx. 38%), significantly improve glucose tolerance (approx. 34%) and decrease body weight gain (approx. 60%) in mice exposed to a high-fat and sucrose diet. Remarkable decrease of body weight gain (approx. 39%) was observed in mice exposed to the standard diet supplemented with APF as well. Thereafter, APF utilisation in various formulations of food and dietary supplements could be a valuable approach to developing dietary strategies for prevention of diet-driven diabetes type 2 as well as obesity management. Positive effects on body weight management, glucose level and glucose tolerance confirmed, also, that the applied technological process provided preservation of biomolecules in their physiologically active form. The efficiency of translated dose obtained using allometric scaling for dose conversion from mice to humans, approx. 10 times lower than a dose of AP used until now, remains to be confirmed in human trials. 

## 5. Patents

Zlatanović, S., Gorjanović, S., Ostojić, S., Micić, D., Pastor, F., Kalušević, A., Laličić-Petronijević, J., 2019a. Method for producing gluten-free flour made of apple pomace. WO2020/027683 06.02.2020. (Published).

## Figures and Tables

**Table 1:** Total phenol (TPC) and flavonoid (TFC) content in apple pomace flour (APF) and apple egg (AE) expressed in mg of gallic acid and quercetin equivalents per gram (mg GAE g^−1^ and mg QE g^−1^).

	TPC FC (mg GAE g^−1^)	TFC (mg QE g^−1^)
APF1	7.7 ± 0.3 ^a^	24.8 ± 1.0 ^bc^
APF2	6.1 ± 0.2 ^b^	27.4 ± 1.4 ^b^
APF3	8.1 ± 0.3 ^a^	34.6 ± 2.2 ^a^
APF4	4.6 ± 0.2 ^c^	18.6 ± 1.0 ^d^
APF5	4.6 ± 0.1 ^c^	21.2 ± 1.3 ^cd^
AE	4.3 ± 0.2 ^c^	12.2 ± 0.7 ^e^
F	163.3	97.1
*p*	<0.001	<0.001

Values are presented as mean ± SD (*n* = 3). Data were subjected to one-way ANOVA (between-subjects factor: type of apple pomace flour; six levels: APF1, APF2, APF3, APF4, APF5 and AE; degree of freedom for both parameters was 5); means with different lowercase superscript in the same column indicate a significant difference of means, according to Tukey’s honest significant difference (HSD) test (*p* < 0.05) among apple pomace flour samples.

**Table 2:** Phenolic compounds identified and quantified (mg kg^−1^) in APF1–5 and AE using HPLC-DAD–MS/MS.

mg kg^−1^	APF1	APF2	APF3	APF4	APF5	AE	*F*	*p*
	**Dihydrochalcones**							
Phloretin	0.77 ± 0.06 ^b^	0.70 ± 0.04 ^bc^	0.98 ± 0.03 ^a^	0.29 ± 0.02 ^d^	0.78 ± 0.00 ^b^	0.63 ± 0.05 ^c^	102.3	<0.001
Phlorizin	215.1 ± 2.5 ^a^	194.5 ± 5.3 ^b^	227.3 ± 1.3 ^a^	112 ± 3.0 ^d^	165.8 ± 1.1 ^c^	158.9 ± 9.4 ^c^	240.4	<0.001
**Total**	**215.9 ± 2.5 ^a^**	**195.2 ± 5.3 ^b^**	**228.3 ± 1.3 ^a^**	**112 ± 3 ^d^**	**166.3 ± 1.3 ^c^**	**159.6 ± 9.4 ^c^**	239.9	<0.001
	**Flavones**							
Luteolin	0.26 ± 0.01 ^a^	0.11 ± 0.01 ^c^	0.15 ± 0.01 ^b^	0.10 ± 0.01 ^c^	0.13 ± 0.01 ^bc^	0.11 ± 0.01 ^c^	89.1	<0.001
Apigenin-7-*O*-glucoside	0.84 ± 0.05 ^b^	0.73 ± 0.02 ^bc^	1.01 ± 0.05 ^a^	0.68 ± 0.03 ^c^	0.47 ± 0.04 ^d^	0.69 ± 0.06 ^c^	49.6	<0.001
Apigenin	0.48 ± 0.00	0.38 ± 0.04	0.46 ± 0.07	0.31 ± 0.01	0.41 ± 0.23	0.43 ± 0.21	0.6	0.675
Chrysin	0.19 ± 0.00 ^b^	0.18 ± 0.00 ^c^	0.22 ± 0.00 ^a^	0.13 ± 0.00 ^d^	0.11 ± 0.00 ^e^	0.12 ± 0.00 ^d^	702.9	<0.001
**Total**	**1.77 ± 0.07 ^a^**	**1.40 ± 0.07 ^ab^**	**1.84 ± 0.11 ^a^**	**1.22 ± 0.03 ^b^**	**1.12 ± 0.28 ^b^**	**1.35 ± 0.26 ^ab^**	6.9	0.003
	**Flavanones**							
Eriodictyol	0.18 ± 0.02 ^b^	0.13 ± 0.02 ^c^	0.26 ± 0.01 ^a^	0.11 ± 0.01 ^c^	0.21 ± 0.02 ^b^	0.21 ± 0.01 ^b^	34.0	<0.001
Naringenin	0.24 ± 0.02 ^a^	0.18 ± 0.03 ^b^	0.21 ± 0.01 ^ab^	0.11 ± 0.00 ^c^	0.17 ± 0.00 ^b^	0.19 ± 0.01 ^ab^	20.2	<0.001
Naringin	0.22 ± 0.01 ^d^	0.57 ± 0.02 ^a^	0.60 ± 0.01 ^a^	0.48 ± 0.02 ^b^	0.35 ± 0.02 ^c^	0.46 ± 0.02 ^b^	195.0	<0.001
**Total**	**0.64 ± 0.01 ^d^**	**0.88 ± 0.01 ^b^**	**1.07 ± 0.01 ^a^**	**0.70 ± 0.03 ^c^**	**0.73 ± 0.04 ^c^**	**0.86 ± 0.00 ^b^**	177.5	<0.001
	**Flavonols and Flavonol Glycosides**							
Quercetin	14.2 ± 3.0 ^a^	10.9 ± 2.4 ^ab^	13.1 ± 3.3 ^ab^	7.2 ± 1.8 ^bc^	4.10 ± 0.74 ^c^	12.7 ± 1.8 ^ab^	8.6	0.001
Quercetin-3-*O*-rhamnoside	121.9 ± 1.3 ^a^	85.3 ± 4.1 ^c^	124.8 ± 1.7 ^a^	114.1 ± 3.9 ^b^	34.1 ± 1.3 ^d^	85.9 ± 1.7 ^c^	512.7	<0.001
Quercetin-3-*O*-galactoside	165.2 ± 3.5 ^a^	158.5 ± 2.7 ^a^	149.9 ± 4.1 ^b^	126.7 ± 3.4 ^c^	121.4 ± 0.6 ^c^	80.8 ± 1.8 ^d^	339.6	<0.001
Rutin	46.93 ± 1.91 ^b^	20.37 ± 0.28 ^e^	23.9 ± 0.2 ^d^	64.86 ± 1.40 ^a^	7.99 ± 0.09 ^f^	34.46 ± 0.84 ^c^	1163.2	<0.001
Isorhamnetin-3-*O*-rutinoside	1.11 ± 0.01 ^a^	0.82 ± 0.02 ^b^	0.41 ± 0.00 ^c^	0.40 ± 0.04 ^c^	0.36 ± 0.04 ^c^	0.10 ± 0.01 ^d^	703.8	<0.001
Isorhamnetin	12.31 ± 0.43 ^b^	17.62 ± 0.31 ^a^	4.05 ± 0.04 ^c^	2.08 ± 0.02 ^d^	1.10 ± 0.01 ^e^	1.16 ± 0.02 ^e^	3037.8	<0.001
Kaempferol	2.46 ± 0.37 ^a^	0.71 ± 0.14 ^bc^	2.82 ± 0.37 ^a^	1.37 ± 0.16 ^b^	0.48 ± 0.02 ^c^	0.62 ± 0.42 ^bc^	37.1	<0.001
Kaempferol-7-*O*-glucoside	0.05 ± 0.01 ^c^	0.03 ± 0.01 ^c^	0.70 ± 0.03 ^b^	1.19 ± 0.12 ^a^	0.11 ± 0.01 ^c^	0.13 ± 0.02 ^c^	251.7	<0.001
**Total**	**363.9 ± 7.8 ^a^**	**257.5 ± 7.3 ^c^**	**328.4 ± 8.6 ^b^**	**341.1 ± 7.4 ^b^**	**129.1 ± 2.5 ^d^**	**261.8 ± 4.4 ^c^**	491.4	<0.001
	**Flavanonols**							
Taxifolin	0.16 ± 0.02 ^d^	0.46 ± 0.01 ^a^	0.33 ± 0.01 ^b^	0.33 ± 0.04 ^b^	0.24 ± 0.01 ^c^	0.21 ± 0.02 ^cd^	86.5	<0.001
	**Hydroxycinnamic acids**							
Caffeic acid	0.33 ± 0.02 ^a^	0.22 ± 0.03 ^b^	0.35 ± 0.05 ^a^	0.23 ± 0.01 ^b^	0.12 ± 0.00 ^c^	0.18 ± 0.00 ^bc^	39.7	<0.001
*p*-Coumaric acid	0.32 ± 0.06 ^c^	0.44 ± 0.09 ^bc^	0.44 ± 0.03 ^bc^	0.76 ± 0.09 ^a^	0.57 ± 0.03 ^b^	0.51 ± 0.05 ^b^	17.5	<0.001
Ferulic acid	23.80 ± 0.28 ^a^	23.43 ± 0.87 ^a^	13.24 ± 0.07 ^d^	23.48 ± 0.21 ^a^	19.48 ± 0.54 ^b^	15.86 ± 0.33 ^c^	281.1	<0.001
Sinapic acid	7.20 ± 0.03 ^a^	4.29 ± 0.19 ^b^	2.97 ± 0.05 ^c^	2.78 ± 0.03 ^cd^	2.60 ± 0.01 ^d^	2.03 ± 0.05 ^e^	1464.3	<0.001
Chlorogenic acid	224.4 ± 9.8 ^b^	214.3 ± 3.4 ^b^	308.3 ± 14.0 ^a^	89.0 ± 9.5 ^e^	185.7 ± 6.2 ^c^	126.6 ± 4.7 ^d^	237.4	<0.001
**Total**	**251.8 ± 10.1 ^b^**	**245.6 ± 3.9 ^b^**	**325.1 ± 14.1 ^a^**	**117.7 ± 9.5 ^e^**	**208.5 ± 6.4 ^c^**	**145.2 ± 4.6 ^d^**	220.9	<0.001
	**Hydroxybenzoic acids**							
Gallic acid	4.53 ± 0.10 ^b^	2.22 ± 0.04 ^e^	4.80 ± 0.12 ^a^	3.20 ± 0.12 ^c^	3.20 ± 0.03 ^c^	2.54 ± 0.03 ^d^	450.4	<0.001
Protocatechuic acid	28.61 ± 1.01 ^a^	7.29 ± 0.55 ^de^	21.15 ± 0.73 ^b^	6.58 ± 0.42 ^e^	17.22 ± 0.24 ^c^	8.69 ± 0.01 ^d^	686.9	<0.001
Ellagic acid	19.70 ± 2.90 ^b^	14.17 ± 0.27 ^b^	24.04 ± 5.64 ^b^	22.88 ± 8.49 ^b^	22.49 ± 2.30 ^b^	75.2 ± 11.8 ^a^	35.5	<0.001
*p*-Hydroxybenzoic acid	2.51 ± 0.10 ^b^	2.15 ± 0.38 ^b^	2.24 ± 0.15 ^b^	2.92 ± 0.53 ^b^	1.16 ± 0.17 ^c^	5.80 ± 0.09 ^a^	89.9	<0.001
**Total**	**55.4 ± 3.4 ^b^**	**25.8 ± 0.5 ^d^**	**52.2 ± 5.2 ^bc^**	**35.6 ± 9.4 ^cd^**	**44.1 ± 1.9 ^bcd^**	**92.3 ± 11.7 ^a^**	35.6	<0.001
	**Coumarins**							
Aesculin	9.38 ± 0.37 ^b^	8.80 ± 0.36 ^bc^	10.67 ± 0.56 ^a^	7.96 ± 0.11 ^c^	9.68 ± 0.28 ^b^	5.53 ± 0.16 ^d^	81.8	<0.001
	**Others**							
Resveratrol	0.16 ± 0.01 ^d^	0.89 ± 0.02 ^a^	0.82 ± 0.01 ^b^	0.24 ± 0.02 ^c^	0.22 ± 0.02 ^c^	0.19 ± 0.03 ^cd^	869.1	<0.001
Pterostilbene	0.19 ± 0.01 ^d^	0.90 ± 0.00 ^a^	0.70 ± 0.01 ^b^	0.35 ± 0.02 ^c^	0.29 ± 0.02 ^c^	0.20 ± 0.05 ^d^	478.3	<0.001
Pinocembrin	0.39 ± 0.01 ^a^	0.32 ± 0.00 ^b^	0.22 ± 0.00 ^e^	0.25 ± 0.00 ^d^	0.29 ± 0.00 ^c^	0.22 ± 0.00 ^e^	938.6	<0.001
	**Total**							
	**899.9 ± 3.9 ^b^**	**736.1 ± 7.2 ^c^**	**948.6 ± 77.0 ^a^**	**619 ± 10 ^e^**	**560.5 ± 4.9 ^f^**	**668.6 ± 19.9^d^**	736.5	<0.001

* Values are presented as mean ± SD (*n* = 3). Data were subjected to one-way ANOVA (between-subjects factor. Type of apple pomace flour; six levels: APF1, APF2, APF3, APF4, APF5 and AE; degree of freedom for all parameters was 5), different superscripts within the same row indicate a significant difference of means, according to Tukey’s HSD test (*p* < 0.05).

**Table 3:** Antioxidant activity of APF1–5 and AE determined by ABTS, DPPH and HPMC as well as Relative Antioxidant Capacity Index (RACI).

	ABTS (mmol TE 100 g^−1^)	DPPH (mmol TE 100 g^−1^)	HPMC (%/mg)	RACI
APF1	10.0 ± 0.7 ^a^	3.8 ± 0.2 ^ab^	1.20 ± 0.04 ^b^	0.63
APF2	9.2 ± 0.9 ^a^	3.3 ± 0.3 ^bc^	1.20 ± 0.04 ^b^	0.35
APF3	9.5 ± 1.0 ^a^	4.5 ± 0.4 ^a^	1.74 ± 0.08 ^a^	1.42
APF4	3.6 ± 0.5 ^b^	2.6 ± 0.2 ^cd^	1.00 ± 0.04 ^c^	−0.68
APF5	3.4 ± 0.3 ^b^	2.9 ± 0.4 ^bcd^	0.90 ± 0.04 ^cd^	−0.68
AE	3.1 ± 0.4 ^b^	2.2 ± 0.5 ^d^	0.86 ± 0.04 ^d^	−1.03
*F*	74.7	17.1	130.5	
*p*	<0.001	<0.001	<0.001	

* Values are presented as mean ± SD (*n* = 3). Data were subjected to one-way ANOVA (between-subjects factor. Type of apple pomace flour; six levels: APF1, APF2, APF3, APF4, APF5 and AE; degree of freedom for all parameters was 5), different superscripts within the same column indicate a significant difference of means, according to Tukey’s HSD test (*p* < 0.05).

**Table 4:** Correlation matrix between TPC, TFC, total phenolics identified by HPLC (TPC–HPLC) and AO activity determined by ABTS, DPPH and HPMC assay.

Variables	DPPH	HPLC	TPC	TFC	HPMC	RACI
ABTS	**0.86**	**0.89**	**0.94**	**0.82**	0.77	**0.93**
DPPH		**0.89**	**0.96**	**0.95**	**0.93**	**0.98**
HPLC			**0.97**	0.75	**0.86**	**0.93**
TPC				**0.86**	**0.88**	**0.97**
TFC					**0.92**	**0.94**
HPMC						**0.95**

Values in bold are different from 0 with a significance level α = 0.05.

**Table 5:** Effect of 150 days of high-fat and sucrose diet with 10 mg of APF supplementation (HFSD+APF10) on glycaemic status food, body weight gain, water, food and energy intake, food efficiency ratio per food (FED1) and per energy intake (FED2), and glucose tolerance in OGTT (AUC) of C57BL/6J mice in comparison to HFSD without APF addition (HFSD) and to the standard pellet rodents diet with (SPRD+APF10) and without the addition of APF (SPRD).

Parameter	HFSD + APF10	HFSD	SPRD	SPRD + APF10	*F*	*p*
Glycemia (mmol/L)	7.6 ± 1.5 ^b^	12.4 ± 2.1 ^a^	8.9 ± 2.3 ^b^	8.9 ± 0.6 ^b^	8.3	0.001
Body weight gain (g)	4.5 ± 1.3 ^b^	11.5 ± 2.3 ^a^	11.1 ± 1.3 ^a^	6.7 ± 1.9 ^b^	22.5	<0.001
Water intake (mL/d)	8.56 ± 0.36	8.44 ± 0.42	8.56 ± 0.36	8.39 ± 0.23	0.28	0.838
Food intake (g/d)	1.99 ± 0.03 ^b^	1.97 ± 0.05 ^b^	2.12 ± 0.03 ^a^	2.10 ± 0.04 ^a^	20.5	<0.001
Energy intake (kcal/d)	15.2 ± 0.2 ^a^	15.0 ± 0.3 ^a^	6.4 ± 0.1 ^b^	6.3 ± 0.1 ^b^	3559.7	<0.001
FER1 ** (g/g)	0.015 ± 0.004 ^b^	0.039 ± 0.008 ^a^	0.035 ± 0.004 ^a^	0.021 ± 0.006 ^b^	19.2	<0.001
FER2 *** (×10^−3^ g/kcal)	2.0 ± 0.6 ^c^	5.1 ± 1.0 ^b^	11.6 ± 1.4 ^a^	7.1 ± 2.0 ^b^	53.3	<0.001
AUC (mM*h)	19.2 ± 2.0 ^b^	29.0 ± 3.6 ^a^	16.3 ± 3.7 ^b^	19.9 ± 4.5 ^b^	14.3	<0.001

* The values are represented as mean ± SD (glycaemia, body weight gain, FER1, FER2 and AUC: *n* = 8; liquid intake, food intake and energy intake: *n* = 21). Data were subjected to one-way ANOVA (between-subjects factor: feed treatment; four levels: HFSD, HFSD + APF10, SPRD and SPRD + APF10, degree of freedom was 3), different superscripts within the same row indicate a significant difference of means, according to Tukey’s HSD test (*p* < 0.05). ** FER1—feed efficiency ratio (weight gain/food intake). *** FER2—feed efficiency ratio (weight gain/energy intake).

## Supplementary Materials

The following are available online at https://www.mdpi.com/2076-3921/9/5/413/s1, Table S1: Retention Time, Parents Ion, Products Ions, Calibration Range, R2, LOD, LOQ for the Investigated Poliphenols.
