# Supplementary material for: Evaluation of Apple Pomace Flour Obtained Industrially by Dehydration as a Source of Biomolecules with Antioxidant, Antidiabetic and Antiobesity Effects
Source: Antioxidants (Basel). 2020 May 12;9(5):413. doi: 10.3390/antiox9050413 (PMC7278621; doi:10.3390/antiox9050413)
Supplement: Supplementary file 1 [file antioxidants-09-00413-s001.pdf]

Table 1. Retention Time, Parents Ion, Products Ions, Calibration Range, R2, LOD, LOQ for the Investigated Poliphenols.

Compounds	Retention Time, *min*	Parent Ion, [M−H]⁻, *m/z*	Product Ions, [M−H]⁻, *m/z* (Collision Energy, *eV*)	Calibration Range, *mg/L*	R^2	LOD, *mg/L*	LOQ, *mg/L*
**Gallic-acid**	2.35	169.032	125.04 (16); 79.11 (31)	0.010 - 2.000	0.9950	0.148	0.493
**Protocatechuic-acid**	4.10	153.003	108.07 (5); 109.10 (15)	0.050 - 2.000	0.9980	0.102	0.339
**Aesculin**	4.86	339.080	133.09 (44); 177.06 (25)	0.008 - 1.800	0.9980	0.093	0.308
***p*-Hydroxybenzoic-acid**	5.20	137.057	93.19 (19); 108.33 (22)	0.110 - 2.200	0.9959	0.161	0.535
**5-O-Caffeoylquinic-acid**	5.31	353.103	191.28 (25)	0.011 - 2.100	0.9993	0.063	0.210
**Caffeic-acid**	5.64	179.004	134.00 (13); 135.00 (16)	0.009 - 1.900	0.9927	0.182	0.606
**Rutin**	6.23	609.197	299.98 (42); 301.20 (32)	0.012 - 2.400	0.9984	0.099	0.331
***p*-Coumaric-acid**	6.39	163.031	93.12 (39); 119.09 (16)	0.010 - 2.000	0.9975	0.110	0.366
**Quercetin-3-O-galactoside**	6.40	463.002	271.01 (44); 300.02 (29)	0.010 - 1.000	0.9942	0.096	0.321
**Ellagic-acid**	6.57	300.980	284.00 (32); 300.04 (30)	0.010 - 1.000	0.9938	0.110	0.367
**Naringin**	6.74	579.241	151.42 (43); 217.36 (33)	0.010 - 2.000	0.9951	0.158	0.527
**Sinapic-acid**	6.75	223.082	149.21 (36)	0.010 - 1.500	0.9968	0.107	0.358
**Kaempferol-7-O-glucoside**	6.83	447.008	255.03 (43); 284.03 (29)	0.003 - 0.500	0.9931	0.047	0.156
**Isorhamnetin-3-O-rutinoside**	6.86	622.827	313.95 (17); 151.21 (27)	0.010 - 2.000	0.9974	0.058	0.193
**Apigenin-7-O-glucoside**	6.92	431.004	239.11 (53); 268.03 (36)	0.001 - 0.200	0.9969	0.007	0.023
**Taxifolin**	7.07	303.020	151.01 (16)	0.010 - 2.000	0.9967	0.057	0.189
**Quercetin-3-O-rhamnoside**	7.15	447.316	300.96 (20); 270.82 (38)	0.010 - 2.000	0.9964	0.068	0.228
**Ferulic-acid**	7.16	193.057	134.00 (18); 178.00 (15)	0.010 - 2.000	0.9902	0.204	0.680
**Phlorizin**	7.47	435.149	273.16 (20); 167.16 (34)	0.010 - 2.000	0.9958	0.039	0.130
**Resveratrol**	7.77	227.060	143.18 (22); 185.04 (22)	0.010 - 2.000	0.9991	0.073	0.242
**Luteolin**	8.17	285.035	133.05 (30); 150.95 (24)	0.025 - 1.000	0.9974	0.062	0.207
**Eriodictyol**	8.57	286.974	150.93 (19); 149.00 (24)	0.010 - 2.000	0.9962	0.098	0.325
**Phloretin**	8.75	273.066	123.26 (26); 167.20	0.010 -	0.997	0.116	0.387

			(19)	2.000	3		
**Quercetin**	8.90	301.026	151.01 (22); 179.00 (20)	0.010 - 2.000	0.9911	0.107	0.358
**Apigenin**	8.92	269.032	117.24 (43); 135.02 (22)	0.010 - 2.000	0.9969	0.095	0.317
**Naringenin**	8.92	271.036	119.10 (25); 151.07 (19)	0.012 - 2.400	0.9971	0.138	0.459
**Kaempferol**	9.02	285.074	211.00 (32); 227.00 (32)	0.025 - 2.000	0.9913	0.252	0.841
**Pterostilbene**	9.22	255.026	196.70 (36)	0.010 - 2.000	0.9968	0.148	0.493
**Isorhamnetin**	10.06	314.989	270.55 (37); 107.00 (34)	0.010 - 2.000	0.9985	0.106	0.354
**Chrysin**	10.72	253.054	119.00 (36); 143.00 (30)	0.010 - 2.000	0.9978	0.052	0.175
**Pinocembrin**	11.75	255.081	213.04 (25); 150.93 (25)	0.010 - 2.000	0.9947	0.077	0.256
